# A new look at the Dynamic Similarity Hypothesis: the importance of swing phase

**DOI:** 10.1242/bio.20135165

**Published:** 2013-08-19

**Authors:** David A. Raichlen, Herman Pontzer, Liza J. Shapiro

**Affiliations:** 1 School of Anthropology, University of Arizona, PO Box 210030, Tucson, AZ 85721-00030, USA; 2 Department of Anthropology, Hunter College, 695 Park Avenue, New York, NY 10065, USA; 3 New York Consortium for Evolutionary Primatology, New York, USA; 4 Department of Anthropology, University of Texas at Austin, 1 University Avenue, Austin, TX 78712, USA

**Keywords:** Biomechanics, Quadrupedalism, Froude, Primate, Locomotion, Spandrels

## Abstract

The Dynamic Similarity Hypothesis (DSH) suggests that when animals of different size walk at similar Froude numbers (equal ratios of inertial and gravitational forces) they will use similar size-corrected gaits. This application of similarity theory to animal biomechanics has contributed to fundamental insights in the mechanics and evolution of a diverse set of locomotor systems. However, despite its popularity, many mammals fail to walk with dynamically similar stride lengths, a key element of gait that determines spontaneous speed and energy costs. Here, we show that the applicability of the DSH is dependent on the inertial forces examined. In general, the inertial forces are thought to be the centripetal force of the inverted pendulum model of stance phase, determined by the length of the limb. If instead we model inertial forces as the centripetal force of the limb acting as a suspended pendulum during swing phase (determined by limb center of mass position), the DSH for stride length variation is fully supported. Thus, the DSH shows that inter-specific differences in spatial kinematics are tied to the evolution of limb mass distribution patterns. Selection may act on morphology to produce a given stride length, or alternatively, stride length may be a “spandrel” of selection acting on limb mass distribution.

## Introduction

The Dynamic Similarity Hypothesis (DSH), first applied to comparative biomechanics by Alexander (Alexander, 1976; Alexander and Jayes, 1983), is one of the most far reaching concepts in animal locomotion and has played an essential role in our understanding of terrestrial locomotor evolution (Vaughan and O'Malley, 2005). The DSH suggests that differences in locomotor mechanics among mammals are due mostly to differences in size, providing a unifying framework for understanding the evolution of locomotion across a wide range of taxa (Alexander and Jayes, 1983). According to the DSH, once size is taken into account, animals that are roughly geometrically similar (dimensions can be made equal by multiplying by a constant) will walk with dynamically similar mechanics (mechanics can be made equal by multiplying by separate constants for spatial, temporal, and kinetic features) when the ratios of inertial and gravitational forces acting on the animals are equal (Alexander and Jayes, 1983), a condition that is met when animals walk at the same Froude number (*Fr*):
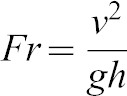
(1)where *v* is velocity (ms^−1^), *g* is gravitational acceleration (9.8 ms^−2^), and *h* is some characteristic length (m). Since the primary inertial force in terrestrial locomotion is generally thought to be the centripetal force acting on an animal as it vaults over its stance leg (e.g. inverted pendulum model) (Cavagna et al., 1977; Cavagna and Margaria, 1966; Donelan and Kram, 1997), *h* is assumed to be hip height (e.g. the length of the strut for the inverted pendulum). Limb length also determines the maximum potential energy of the body in the inverted pendulum model, so its use in Eqn 1 suggests that ratios of potential and kinetic energy are equal at equal *Fr*s (Biewener, 2003). Since gait parameters change as a consequence of both the speed and size of an animal, the DSH has proven invaluable in allowing researchers to compare organisms of different size walking at different speeds by providing a size-corrected speed (*Fr*) against which gait parameters are compared. In this way, the DSH has played an essential role in the development of comparative and evolutionary biomechanics (Alexander and Jayes, 1983; Alexander and Maloiy, 1984; Bennett, 1987; Cavagna et al., 1983; Demes et al., 1990; Donelan and Kram, 1997; Gatesy and Biewener, 1991; Kram et al., 1997; McGeer, 1990; Moretto et al., 1996; Raichlen, 2005a; Raichlen, 2006; Shapiro and Young, 2012; Zijlstra et al., 1996), in studies of clinical human gait (Vaughan et al., 2003; Vaughan and O'Malley, 2005), to predict locomotor mechanics on other planets (Minetti, 2001a; Minetti, 2001b; Raichlen, 2008), and in reconstructions of locomotion in fossil humans and dinosaurs (Alexander, 1976; Alexander, 1984; Dingwall et al., 2013; Raichlen et al., 2008; Vaughan and Blaszczyk, 2008).

One key aspect of the DSH, and one that drives several aspects of animal locomotor mechanics and energetics, is the requirement that animals walk with dynamically similar stride lengths relative to size at similar *Fr*s (Alexander and Jayes, 1983). However, two issues have arisen in studies examining stride length and the DSH. First, experiments with a gravity altering treadmill suggest that human stride lengths are not equal at the same *Fr*s when gravity is changed (Donelan and Kram, 1997). Second, as Alexander and Jayes originally noted (Alexander and Jayes, 1983), some groups of mammals (non-cursorial quadrupeds and non-human primates) seem to walk with longer relative stride lengths compared to other mammalian quadrupeds at similar *Fr*s (Alexander and Maloiy, 1984; Demes et al., 1990; Raichlen, 2004; Raichlen, 2005a; Raichlen, 2005b; Raichlen, 2006). This violation of a key element of the DSH requires a more detailed examination to determine whether it indeed describes mammalian locomotion in the general way it is applied across so many disciplines. Moreover, violations of the DSH may help us better understand the adaptive value of locomotor mechanics, since, for example, walking with relatively long strides at a given speed may allow for safer locomotion on unstable substrates (e.g. small narrow branches), or may contribute to energetically economical walking and running (Demes et al., 1990; Pontzer, 2007a). Thus, understanding why animals deviate from dynamically similar stride lengths could provide a unique window into the evolution of mammalian locomotion.

Here, we suggest that violations of the DSH are due to a focus on stance phase dynamics, despite evidence that limb motion during swing phase plays a major role in determining gait characteristics. Locomotor kinematics are influenced to some extent by a combination of the natural period of the limb swinging as a suspended pendulum and by the muscle force required to drive the limb above or below its natural period (Holt et al., 1990; Kuo, 2001; Kuo, 2002; McGeer, 1990; McMahon, 1984; Mochon and McMahon, 1980; Preuschoft, 2004; Preuschoft and Günther, 1994; Raichlen, 2004; Raichlen, 2008). Since mass distribution determines a limb's natural period, more distal limb centers of mass (COM) lead to longer swing durations, and therefore longer strides and lower stride frequencies (Holt et al., 1990; Preuschoft and Günther, 1994; Raichlen, 2004; Raichlen, 2008).

If we shift the focus of the DSH from stance to swing phase, then the inertial force used to calculate the Froude number may be related to the limb swinging as a suspended pendulum, and therefore *h* in Eqn 1 would be limb COM position rather than hip height. This hypothesis was indirectly tested using a kinematic model to show that the effects of gravity on limb swing account for changes in human stride length under reduced gravity conditions (Raichlen, 2008). In addition, Leurs et al. showed in a novel experiment that increasing limb length in humans through use of stilts results in a change in relative stride length at a given Froude number (Leurs et al., 2011), suggesting that limb geometry, rather than limb length alone, plays an important role in generating dynamic similarity. Here, we test this hypothesis directly using experimental data from a range of taxa and predict that variation in limb mass distribution will account for deviations from dynamically similar stride lengths across mammals. If supported, this hypothesis would resolve violations of the DSH and would suggest that mammalian locomotion is governed to a large extent by swing phase dynamics.

## Materials and Methods

We compared *Fr* (Eqn 1) and relative stride length (rSL; stride length divided by characteristic length, *h*) in a variety of species using two characteristic lengths (*h*): hip height (measured as the perpendicular distance from greater trochanter to the ground) and hindlimb COM position. Locomotor and morphological data included here are from previous studies of comparative biomechanics in the following taxa (Pontzer, 2007b; Raichlen, 2005a; Raichlen, 2005b; Raichlen, 2006; Shapiro and Raichlen, 2006; Shapiro and Raichlen, 2005; Sockol et al., 2007): infant *Papio cynocephalus* (baboons; *n* = 4), *Pan troglodytes* (chimpanzees; *n* = 5), *Canis familiaris* (dogs; *n* = 4), *Capra hircus* (goats; *n* = 4), and *Homo sapiens* (humans; *n* = 5). In a second experiment, we altered human hindlimb COM positions by strapping weights to their ankles (0.75 kg on each ankle).

Two methods were used to calculate limb COM positions. For humans, dogs and goats, hindlimb lengths, and hindlimb segment lengths, were measured using measuring tape, and hindlimb COM positions were calculated from equations in Winter's paper for humans (Winter, 1990), and Myers and Steudel for dogs and goats (Myers and Steudel, 1997). For humans, COM positions were calculated by summing limb segment COM positions, weighted by segment mass, relative to the hip joint (with the foot treated as a point mass at the distal end of the shank). We use hindlimb COM positions and lengths here because the natural period of forelimbs and hindlimbs are generally similar in quadrupeds and use of hindlimb remains consistent with previous studies of the DSH (Alexander and Jayes, 1983; Myers and Steudel, 1997; Raichlen, 2004). Ankle weights were treated as point masses at the distal end of the shank. For chimpanzees and baboons, geometric models were used to calculate limb COM positions (Raichlen, 2004). External measurements were taken of limb segment lengths and circumferences, and geometric models were used to calculate segment COM positions (Raichlen, 2004). As in humans, non-human primate hindlimb COM positions were calculated by summing limb segment COM positions, weighted by segment mass, relative to the hip joint.

Locomotor data were captured from overground locomotion in baboons and from treadmill locomotion in all other taxa. These data were from previous studies of locomotor mechanics in these taxa (Raichlen, 2005a; Pontzer, 2007b; Sockol et al., 2007). Chimpanzees walked on a motorized treadmill while they were filmed with high-speed digital video (Redlake®, San Diego, CA, 125 Hz). Frame-by-frame coordinates of hindlimb joint centers (marked with non-toxic white paint) were calculated using a Matlab image analysis program (Hedrick, 2008). Dogs and goats walked on a motorized treadmill and their joints were marked using small reflective markers adhered to the skin via double-sided tape and tracked using a high-speed infrared camera system (Qualysis®, Gothenburg, Sweden, 200 Hz). Frame-by-frame coordinates of each joint center were calculated using Qualysis proprietary software. Baboons walked in a clear plastic tunnel and their joints were marked with spherical reflective markers. Markers were tracked using an infrared motion analysis system (Vicon®, Oxford, UK, 60 Hz) and frame-by-frame coordinates of joint centers were calculated using Vicon proprietary software. Analyses were restricted to the sagittal plane. For treadmill trials, stride lengths were calculated as the product of velocity (e.g. treadmill velocity) and stride duration. For baboons, stride lengths were calculated as the distance traveled by the hindfoot between two successive footfalls, and velocity was calculated as stride length divided by stride duration.

Human subjects walked on a treadmill at three speeds (1, 1.5, and 2 ms^−1^), and markers on their limbs joints were tracked using a high-speed infrared camera system (Qualysis®, 200 Hz). In addition to normal walking at three speeds, human subjects walked at these same speeds while wearing ankle weights (0.75 kg on each ankle) to increase the distance of the hindlimb COM from their hip joints.

### Data analysis

For most analyses, ANCOVAs were used to compare relative stride lengths across taxa with Froude number as the covariate. However, the slopes describing the relationship between relative stride length and *Fr* calculated with COM as the characteristic length in infant baboons are not parallel to those of other taxa (see Results section), so ANCOVA is not an appropriate test (Raubenheimer and Simpson, 1992; Sokal and Rohlf, 1995). Differences in rSLs between infant baboons and other taxa were tested using a method developed by Tsutakawa and Hewett (Tsutakawa and Hewett, 1978). This method allows for the comparison of two non-parallel regression lines over a finite range (Tsutakawa and Hewett, 1978; Sokal and Rohlf, 1995). Thus, infant baboon rSLs were compared to other taxa over the range in which the datasets overlap (Tsutakawa and Hewett, 1978).

### Comparative mass distribution dataset

We used comparative data for a broad sample of mammals to determine whether mammalian limb mass distributions explain the broad relative stride length differences across taxa found by Alexander and Jayes (Alexander and Jayes, 1983). We compared limb mass distribution in different taxa by calculating the mass of distal elements (leg and foot or forearm and hand) relative to mass of the proximal element (thigh or arm) using data compiled from the literature (Buchner et al., 1997; Grand, 1977; Grand, 1983). Using data from these studies, we grouped quadrupeds into three general categories: cursorial (*Canis, Equis, Felis*), non-cursorial (*Metachirus, Tupaia, Monodelphis, Philander, Caluromys, Didelphis, Marmosa*), and primates (*Papio, Galago, Macaca, Aotus, Ateles, Cebus, Nycticebus, Perodicticus, Alouatta*).

## Results

**Fig. 1.**
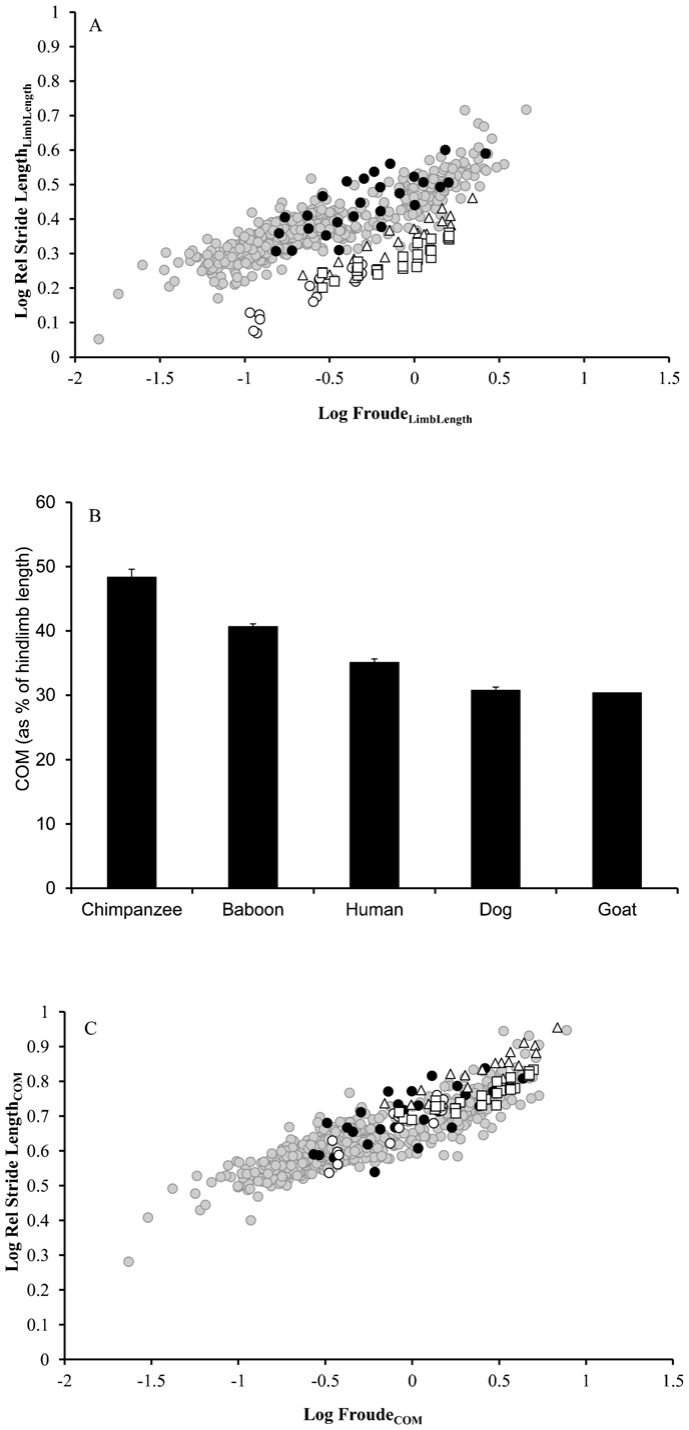
Comparison of stride lengths in mammals. (**A**) Froude numbers and relative stride lengths calculated with hip height. Gray circles are infant baboons, closed circles are chimpanzees, open circles are humans, open triangles are dogs, and open squares are goats. (**B**) Hindlimb COM positions as a percentage of hip height. (**C**) Froude numbers and relative stride lengths calculated with hindlimb COM.

Chimpanzees and baboons use long hindlimb strides relative to limb length at a given *Fr* compared to non-primates and humans (ANCOVA *P*<0.05 with *Fr* as the covariate; Fig. 1A). When hindlimb COM position (Fig. 1B) is used to calculate *Fr* and rSL, differences in rSL between primates and non-primates/humans disappear (Fig. 1C). Chimpanzee rSLs do not differ significantly from those of non-primates and humans (ANCOVA *P* = 0.24 with *Fr* as the covariate; Fig. 1C). Over the range of overlapping data, infant baboon rSLs do not differ significantly from those of other taxa (Tsutakawa and Hewett Test *P* = 0.23).

**Fig. 2.**
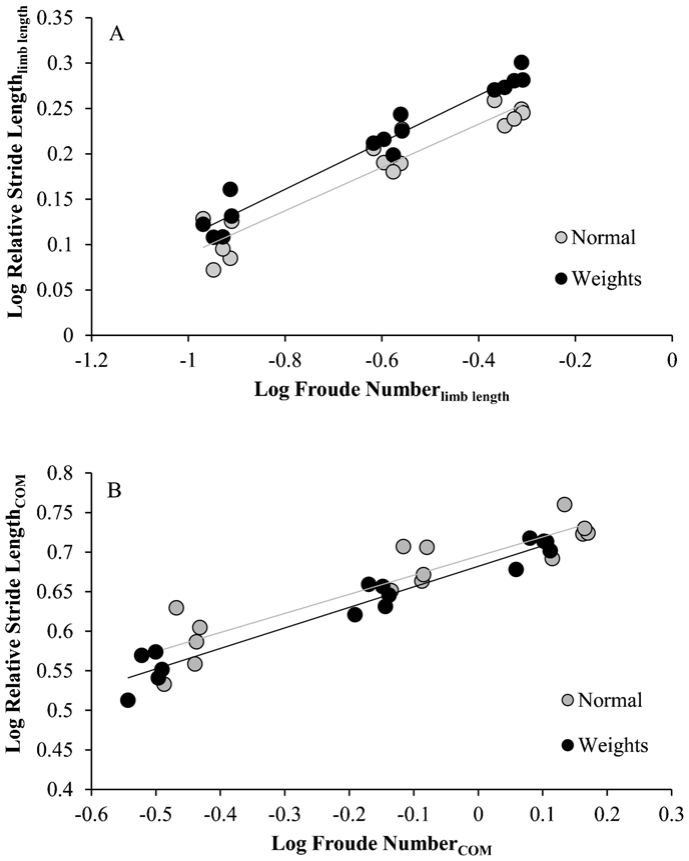
Stride lengths in humans with experimentally altered hindlimb COM positions. (**A**) Stride length calculated using hip height in humans walking normally (gray circles) and walking with 0.75 kg weights on each ankle (black circles). (**B**) Stride length calculated using hindlimb COM position in humans walking normally (gray circles) and walking with 0.75 kg weights on each ankle (black circles).

A similar effect is found in the human sample comparing stride lengths in normal walking and walking with ankle weights. In human subjects wearing ankle weights, rSLs were significantly longer compared to normal trials when analyzed using limb length as the characteristic length (Fig. 2A; ANCOVA *P* = 0.001). When *Fr* and rSL were calculated using hindlimb COM as the characteristic length, differences are no longer significant (Fig. 2B; ANCOVA *P* = 0.07).

**Fig. 3.**
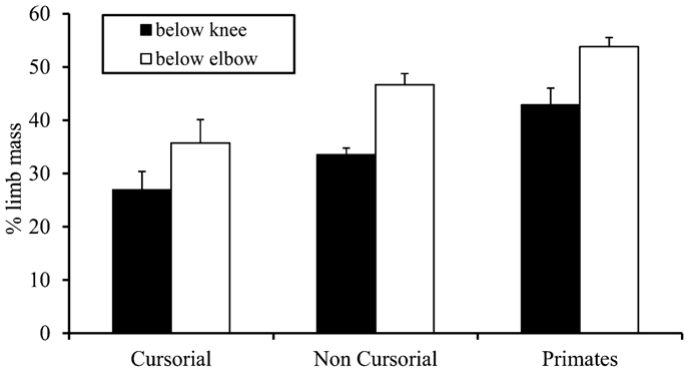
Limb mass distribution in primates compared to cursorial and non-cursorial mammals. Values represent the mean percentage of limb mass below the knee (black) or elbow (white). Groups are: cursorial (*Canis, Equis, Felis*), non-cursorial (*Metachirus, Tupaia, Monodelphis, Philander, Caluromys, Didelphis, Marmosa*), and primates (*Papio, Galago, Macaca, Aotus, Ateles, Cebus, Nycticebus, Perodicticus, Alouatta*). Data are taken from Grand, and Buchner et al. (Grand, 1977; Grand, 1983; Buchner et al., 1997).

To determine whether patterns of limb COM position broadly explain differences in stride lengths among mammals, we also compared relative mass distributions in groups that differ in rSL (i.e. cursorial mammals, non-cursorial mammals, and primates). As described in previous studies (Alexander and Maloiy, 1984), primates have relatively longer strides than other mammalian quadrupeds, and non-cursorial mammals have relative stride lengths that fall between primates and cursorial mammals. Differences in limb mass distribution follow differences in rSL, with primates having the most distally heavy limbs (both fore- and hindlimbs) and cursorial mammals having the most distally light limbs (Fig. 3).

## Discussion

This examination of the DSH reveals the importance of limb mass distribution and swing phase dynamics in driving differences in locomotor mechanics, providing a key morphological link to variation in mammalian gait. While most studies of the DSH focus on stance phase mechanics and the importance of the inverted pendulum model of walking, our study suggests that inertial forces acting on the limb as a suspended pendulum during swing phase may be equally important. Thus, some tests of the DSH may fail (e.g. Donelan and Kram, 1997; Leurs et al., 2011; Kramer and Sylvester, 2013) because they do not take into account the effects of mass distribution on the inertial forces governing swing phase.

In addition, our results suggest that the continuum of limb mass distributions among mammals may broadly explain deviations from traditionally calculated dynamic similarity (e.g. Alexander and Jayes, 1983; Alexander and Maloiy, 1984). Since stride length determines many other locomotor parameters, including speed and energy costs (Gray, 1944; Jones and Lindstedt, 1993; Pontzer, 2007a), understanding the underlying causes of stride length variation is a key to determining how and why animals walk the way they do. For example, more cursorial mammals have relatively light distal limbs and proximal COM positions, while non-cursorial non-primates have relatively heavier distal limbs, and primates have the heaviest distal limbs used for strong grasping in arboreal settings (Preuschoft and Günther, 1994; Raichlen, 2004; Raichlen, 2005a; Raichlen, 2005b; Raichlen, 2006). This morphological continuum roughly follows the stride length differences in these taxa (Alexander and Jayes, 1983; Alexander and Maloiy, 1984), where cursorial mammals have the relatively shortest strides and non-cursors have intermediate relative stride lengths compared to cursors and primates.

We note that our examination of comparative muscle mass distribution does not take into account the effects of limb joint flexion during swing phase on COM position. For example, non-cursorial mammals may flex their elbows and knees to a greater extent during swing phase, shortening their characteristic lengths. It is also possible that under some circumstances, such as walking on tree branches, some taxa may hold their limbs more extended, increasing their COM positions and leading to even longer strides. Thus, more work is required to fully understand the dynamic position of the limb COM during walking in various taxa, and how swing phase mechanics influences stride length.

However, given our results, we suggest that, in general, more distal COM positions seem to drive variation in stride lengths across taxa, and the evolution of stride length variation is likely tied to morphological variation. Selection may act on morphology to produce a given stride length (e.g. concentrate limb mass proximally in cursorial mammals), or alternatively, stride length may be a “spandrel” of selection acting on limb muscle mass distribution (e.g. selection for grasping hands and feet in arboreal taxa) (Gould and Lewontin, 1979).

In sum, this study suggests a fundamental change in how we examine mammalian locomotor variation and evolution. Instead of focusing solely on the inverted pendulum model of body COM movement, we believe that incorporation of the suspended pendulum model of swing phase will generate a more complete view of mammalian locomotion. This swing phase view of comparative biomechanics is especially important when comparing taxa that differ greatly in limb mass distribution. Examined this way, the DSH allows us to determine clear links between anatomy and locomotor mechanics, and helps us generate broader explanations for the evolution of mammalian locomotor patterns.
